# The Rationale for Using Rifabutin in the Treatment of MDR and XDR Tuberculosis Outbreaks

**DOI:** 10.1371/journal.pone.0059414

**Published:** 2013-03-19

**Authors:** Frederick A. Sirgel, Robin M. Warren, Erik C. Böttger, Marisa Klopper, Thomas C. Victor, Paul D. van Helden

**Affiliations:** 1 DST/NRF Centre of Excellence for Biomedical TB Research/MRC Centre for Molecular and Cellular Biology, Division of Molecular Biology and Human Genetics, Faculty of Health Science, Stellenbosch University, Stellenbosch, South Africa; 2 Institut für Medizinische Mikrobiologie, Universität Zürich, Zürich, Switzerland; 3 Nationales Zentrum für Mykobakterien, Universität Zürich, Zürich, Switzerland; University of Padova, Medical School, Italy

## Abstract

Genetically related *Mycobacterium tuberculosis* strains with alterations at codon 516 in the *rpoB* gene were observed amongst a substantial number of patients with drug resistant tuberculosis in the Eastern Cape Province (ECP) of South Africa. Mutations at codon 516 are usually associated with lower level rifampicin (RIF) resistance, while susceptibility to rifabutin (RFB) remains intact. This study was conducted to assess the rationale for using RFB as a substitution for RIF in the treatment of MDR and XDR tuberculosis outbreaks. Minimum inhibitory concentrations (MICs) of 34 drug resistant clinical isolates of *M tuberculosis* were determined by MGIT 960 and correlated with *rpoB* mutations. RFB MICs ranged from 0.125 to 0.25 µg/ml in the 34 test isolates thereby confirming phenotypic susceptibility as per critical concentration (CC) of 0.5 µg/ml. The corresponding RIF MICs ranged between 5 and 15 µg/ml, which is well above the CC of 1.0 µg/ml. Molecular-based drug susceptibility testing provides important pharmacogenetic insight by demonstrating a direct correlation between defined *rpoB* mutation and the level of RFB susceptibility. We suggest that isolates with marginally reduced susceptibility as compared to the epidemiological cut-off for wild-type strains (0.064 µg/ml), but lower than the current CC (≤0.5 µg/ml), are categorised as intermediate. Two breakpoints (0.064 µg/ml and 0.5 µg/ml) are recommended to distinguish between susceptible, intermediate and RFB resistant strains. This concept may assist clinicians and policy makers to make objective therapeutic decisions, especially in situations where therapeutic options are limited. The use of RFB in the ECP may improve therapeutic success and consequently minimise the risk of ongoing transmission of drug resistant *M. tuberculosis* strains.

## Introduction

Mutations within an 81-bp fragment of the *rpoB* gene that encodes the ß subunit of DNA-dependent RNA polymerase are responsible for RIF resistance in *M. tuberculosis* [1]–[4]. This domain is found between *rpoB* codons 507 and 533 and is referred to as the Rifampicin Resistance Determining Region (RRDR). More than 95% of RIF-resistant isolates have been shown to possess mutations within the RRDR of the *rpoB* gene [1]–[7]. Mutations in the RRDR at codons 531, 526 and 513 are generally associated with high-level RIF-resistance [2]–[4]. In contrast, amino acid substitutions resulting from specific changes at codons 511, 514, 515, 516, 518, 521, 522 and 533 are correlated with lower levels of RIF-resistance [2], [8], [9]. Mutations in the *rpoB* gene that confer high-level RIF-resistance (MICs, ≥32 µg/ml) in *M. tuberculosis* have been associated with cross-resistance to RFB (MIC, ≥4.0 µg/ml) [2], [8], [9]. Conversely, isolates that exhibit lower levels of RIF-resistance MICs (≤16 µg/ml) were found to remain phenotypically susceptible to RFB based on a CC of 0.5 µg/ml [2], [8], [9]. Single nucleotide polymorphisms (SNPs) outside the RRDR near the beginning of the *rpoB* gene have also been described to be associated with RIF resistance [5]. This region has recently been suggested for inclusion as an additional target for the detection of cross-resistance between RIF and RFB [5]. High-level RIF-resistance is almost always encountered in clinical practice, while mutants with lower levels of resistance are less frequently reported [10]. The clinical impact of low-level RIF-resistance in multidrug-resistant (MDR) tuberculosis (TB) and extensive drug-resistant (XDR) TB, collectively referred to as M(X)DR-TB, is less well-studied and understood.

Epidemiological data based on molecular methods demonstrated that large numbers of M(X)DR patients in the Eastern Cape Province (ECP) of South Africa are infected with similar *M. tuberculosis* isolates of the atypical Beijing genotype [11]. These isolates had comparable sequence alterations in the *inhA* promoter, *katG*, *rpoB*, *pncA, emb*B and *rrs* (500 and 1400 regions) genes which mediate isoniazid (INH), RIF, pyrazinamide, ethambutol (EMB), streptomycin and amikacin/kanamycin resistance, respectively [11]. These similarities suggest genotypic clustering of circulating strains which are likely responsible for wide-spread transmission of M(X)DR-TB in the ECP. Of particular interest is the high proportion (77%) of the atypical Beijing isolates harbouring SNPs at codon 516 in the *rpoB* gene [11], which is expected not to mediate RFB-resistance [2], [9], [12]. Based on these observations, it was decided to investigate the possibility of using RFB as a substitute for RIF to treat these M(X)DR TB-infected patients. Hence, our objectives: (i) to correlate the MICs of RIF and RFB in a subset of M(X)DR *M. tuberculosis* isolates (ii) to analyse the MIC data to establish whether cross-resistance occurs between RIF and its analogue RFB and, (iii) to translate the gained knowledge into clinical practice for further assessment concerning RFBs potential to improve clinical outcome.

## Materials and Methods

### Clinical Isolates

Amongst a collection of 342 M(X)DR *M. tuberculosis* clinical isolates obtained from patients resident in the ECP, South Africa, 217 (63%) were previously characterised as members of the atypical Beijing lineage [11]. The isolates were cultured from patients with pulmonary TB, but data on the HIV (human immunodeficiency virus) status and the clinical history of the patients were not available. Seventy seven percent (168/217) of the atypical Beijing strains harboured a mutation at codon 516 in the *rpoB* gene and 151/168 (90%) of these had an Asp516Val (GAC→GTC) alteration. A convenience sample of 34/168 isolates (Table 1) with SNPs in the *rpoB* gene at codon 516 was selected for this study. The selection was based on genotypic data which reflect a high degree of homology between the atypical Beijing isolates [11]. All the isolates had known sequence alterations in the *inhA* promoter, *katG* (315), *rpoB* (516), *pncA* (Ins172) and *embB* (306) genes [11]. In addition, high confidence drug-resistance conferring mutations were observed in the *rrs* (500 and 1400 regions) and *gyrA* genes. H37Rv (ATCC 27294) and 27 *M. tuberculosis* clinical isolates were included for quality control purposes. Two of the isolates were genotypically and phenotypically resistant and 26 susceptible to RIF (Table 1).

**Table 1:** MICs and relative resistance of rifampicin and rifabutin in *M. tuberculosis.*

Genotype	*rpoB*	Rifampicin		Rifabutin	
	Mutants (n)	MIC µg/ml	bRR	MIC µg/ml	RR
**Atypical Beijing**	D516T (1)	5.0	10	0.125	2
	D516S (4)	5.0–15	10–30	0.125–0.25	2–4
	D516V (29)	10–15	20–30	0.125–0.25	2–4
**Undetermined**	aWild-type (26)	≤0.5	–	≤0.06	–
**Typical Beijing**	S531L (1)	>10	>20	>1.0	>16
**Atypical Beijing**	Q510P (1)	>10	>20	>1.0	>16

a Twenty-five clinical isolates with unknown genotype plus one H37Rv strain were included as controls.

b RR indicates relative resistance: Mutant MIC/Wild-type MIC.

### MIC Determinations

MICs for all selected isolates were determined for RIF and RFB by quantitative drug susceptibility testing (QDST) in BACTEC MGIT 960 eXtended individual Susceptibility Testing (TB eXiST) for EpiCenter ™ V5.75A, (BD Bioscience, Erembodegem, Belgium) as previously described [13]. The drugs were purchased from Sigma-Aldrich, South Africa. RIF and RFB were dissolved in dimethyl sulfoxide and then diluted in sterile distilled water. Stock solutions of each drug were prepared at concentrations that were at least 84 times higher than the highest test concentration used. The stock solutions were filter sterilized and small aliquots were then stored at −80°C. The MICs for RIF were determined at 0.5 µg/ml, 1.0 µg/ml, 10.0 µg/ml, 15.0 µg/ml and 20.0 µg/ml and for RFB at 0.03 µg/ml, 0.06 µg/ml, 0.125 µg/ml, 0.25 µg/ml, 0.5 µg/ml and 1.0 µg/ml. CCs of 1.0 µg/ml and 0.5 µg/ml were used to determine the susceptibilities of the strains against RIF and RFB, respectively [14]. The relative resistance (RR) of the isolates against the drugs was measured by: Mutant MICs/Wild-type MICs.

## Results

The MICs for RIF and RFB as determined in this study are summarised in Table 1. The selected 34 isolates possessed either an Asp516Tyr (n = 1), Asp516Ser (n = 4) or an Asp516Val (n = 29) *rpoB* mutation. The RIF MICs for these isolates were 5 µg/ml (n = 2) and 10–15 µg/ml (n = 32), which clearly distinguished them from H37Rv and the 25 wild-type strains which displayed MICs of ≤0.5 µg/ml. The level of RIF resistance were thus 5- to 15- fold above the CC, but much lower than those generally displayed by strains harbouring mutations at codons 526 and 531 (MICs, ≥50 to ≥250 µg/ml) [7], [10]. However, the corresponding MICs for RFB ranged between 0.125 and 0.25 µg/ml, which was a 1/4 to a 1/2 of its CC of 0.5 µg/ml [14]. The MICs for the 26 wild-type strains ranged from ≤0.03 to 0.06 µg/ml for RFB. Two mutant control strains with SNPs at codons Ser531Leu and Gln510Pro in the *rpoB* gene had MICs of >10 µg/ml and >1.0 µg/ml for RIF and RFB, respectively. These susceptibility levels were well within the resistance ranges of the respective drugs.

## Discussion

The 34 clinical isolates were phenotypically susceptible to RFB as per CC, despite of their resistance to RIF and the presence of SNPs at codon 516 in the *rpoB* gene (Table 1). However, a shift in the RFB MICs, from ≤0.03–0.06 µg/ml for wild-type strains to 0.125–0.25 µg/ml for the mutant isolates was observed. The corresponding MIC shift for RIF was from ≤0.5 µg/ml to 5.0–15.0 µg/ml. Based on these findings, the relative resistance of the drugs (Table 1) shows that RFB was less affected by the mutations at codon 516 in the *rpoB* gene as compared to RIF [2], [8], [9]. The decreased susceptibility to RFB may not predict clinical resistance, but indicate that mutations at codon 516 in the RRDR are associated with incomplete cross-resistance between RIF and RFB. More recently, an epidemiological cut-off (ECOFF) concentration of 0.064 µg/ml was proposed for RFB based on the Middlebrook 7H10 dilution method [15]. The ECOFF is defined as the highest concentration within the MIC distribution of wild-type strains (i.e. isolates lacking resistance mechanisms) [15]. A breakpoint for RFB, based on clinical evidence has not yet been established. According to the CC (0.5 µg/ml) endorsed by the World Health Organization [14], our results suggest that a substantial proportion M(X)DR TB patients in the ECP may benefit from a treatment regimen that substitute RIF for RFB. This strategy is feasible only if the strains that remain susceptible to RFB are readily detectable. Molecular assays are therefore useful to assist culture-based drug susceptibility testing (DST) in identifying isolates with specific mutations that are associated with RIF-resistance, while they remain susceptible to RFB. The GenoType**®** MTBD*plus* assay (Hain LifeScience GmbH, Nehren, Germany) is designed to detect most of the mutations that confer RIF- and INH- resistance and has been suggested to be an important tool to define RFB susceptibility [16]. However, molecular assays with enhanced discriminating capacity are needed for identifying mutations that confer low-level or incomplete cross-resistance to analogue drugs. This information is crucial, particularly for the rifamycins, INH, the fluoroquinolones and the injectable drugs as the definition of MDR- and XDR- TB is based on these compounds [17]. Furthermore, mutations outside the RRDR, in the beginning of the *rpoB* gene, have also been associated with resistance to both RIF and RFB, while others confer resistance only to RIF [5]. Molecular assays that exclude this region and only target the RRDR in the *rpoB* gene may give a genotypic susceptible result which does not match the resistance phenotype [5].

Previous studies reported on borderline RIF-resistance, missed by standard DST [10], [18]. The particular isolates possessed *rpoB* mutations that were associated with low-level RIF-resistance as confirmed by their MICs [10], [18]. In another study, isolates from an outbreak of MDR-TB were identified with low-level RIF-resistance [9]. All the isolates harboured an Asp516Tyr mutation in the *rpoB* gene and displayed MICs of 0.5–2 µg/ml for RIF and 0.2–0.5 µg/ml for RFB [9]. These studies suggest that low-level RIF-resistance can be overcome by the use of higher RIF doses or alternatively, by replacing RIF with RFB [9], [10]. A retrospective study in South Korea showed an 85.7% (12/14) treatment success in patients with RFB-susceptible MDR-TB infections who received RFB as an additional drug [19]. In our study, the MIC distribution for RFB was above the ECOFF, but below the standard CC. Using of the ECOFF as clinical breakpoint for RFB as recently suggested [15] may be misleading as strains with decreased susceptibility that remain treatable may be overlooked. *M. tuberculosis* strains with moderate decreases in susceptibility to RFB should rather be classified as intermediate. Clinical isolates in this category can be distinguished from those that are clearly susceptible or resistant by introducing a second breakpoint and/or by the use of a molecular assay. Our results and existing data [9], [10], [14], [15], [18] suggest an intermediate classification that encompasses MICs above the ECOFF (0.064 µg/ml), but below or equal to the current CC (0.5 µg/ml). The peak serum concentration of RFB at a single dosage of 300 mg ranges from 0.4 µg/ml to 0.6 µg/ml [20]. Increased RFB dosages may not be an option to increase these levels due to possible toxicity issues. However, the pharmacokinetics of RFB are in part misleading as its blood levels do not reflect the concentrations that are attained in infected cells and tissues where the drug tends to accumulate [20]. Furthermore, a twofold reduction in the MICs of both RFB and EMB has previously been indicated owing to synergy between these two drugs when used together [20].

Our study reinforces the notion that the heterogeneous MIC levels observed in drug resistant *M. tuberculosis* strains may have important therapeutic implications [21]–[24]. Of particular relevance is the presence of mutations that confer low-level drug-resistance as it offers possibilities for more effective treatment of drug resistant TB [21]–[24]. Knowledge of incomplete cross-resistance between the rifamycins and the identification of isolates with intermediate RFB susceptibility should assist clinicians to make objective therapeutic decisions regarding its potential use in M(X)DR-TB treatment-regimens. Designing a treatment regimen for M(X)DR-TB is challenging and the substitution of one or two drugs in a failing regimen must be done with caution to avoid the development of further resistance. Relevant clinical studies are thus imperative to establish appropriate RFB-based regimens that warrant favourable clinical outcome.
