# Correction: The Rationale for Using Rifabutin in the Treatment of MDR and XDR Tuberculosis Outbreaks

**DOI:** 10.1371/journal.pone.0134190

**Published:** 2015-07-21

**Authors:** Frederick A. Sirgel, Robin M. Warren, Erik C. Böttger, Marisa Klopper, Thomas C. Victor, Paul D. van Helden

There is an error in Table 1. An AspTyr mutation is wrongly abbreviated as D516T (1). It should be listed as D516Y (1). Please see the corrected Table 1 here.

**Table 1:** MICs and relative resistance of rifampicin and rifabutin in *M*. *tuberculosis*.

Genotype	*rpoB*	Rifampicin		Rifabutin	
	Mutants (n)	MIC μg/ml	^b^RR	MIC μg/ml	RR
**Atypical Beijing**	D516Y (1)	5.0	10	0.125	2
	D516S (4)	5.0–15	10–30	0.125–0.25	2–4
	D516V (29)	10–15	20–30	0.125–0.25	2–4
**Undetermined**	^a^Wild-type (26)	≤0.5	-	≤0.06	-
**Typical Beijing**	S531L (1)	>10	>20	>1.0	>16
**Atypical Beijing**	Q510P (1)	>10	>20	>1.0	>16

^**a**^Twenty-five clinical isolates with unknown genotype plus one H37Rv strain were included as controls.

^**b**^RR indicates relative resistance: Mutant MIC/Wild-type MIC.
